# SONOICE! a Sonar–Voice dynamic user interface for assisting individuals with blindness and visual impairment in pinpointing elements in 2D tactile readers

**DOI:** 10.3389/fresc.2024.1368983

**Published:** 2024-08-23

**Authors:** Gaspar Ramôa, Vincent Schmidt, Thorsten Schwarz, Rainer Stiefelhagen, Peter König

**Affiliations:** ^1^ Research Department, Inventivio GmbH, Nürnberg, Germany; ^2^ Institute of Cognitive Science, Osnabrück University, Osnabrück, Germany; ^3^ ACCESS@KIT, Karlsruhe Institute of Technology, Karlsruhe, Germany; ^4^ HCI@KIT, Karlsruhe Institute of Technology, Karlsruhe, Germany; ^5^ Institute of Neurophysiology & Pathophysiology, University Medical Center Hamburg-Eppendorf, Hamburg, Germany

**Keywords:** pinpoint navigation, user interface, sonification and speech interfaces, 2D tactile readers, access to graphical information, blind and visually impaired, assistive technology

## Abstract

Pinpointing elements on large tactile surfaces is challenging for individuals with blindness and visual impairment (BVI) seeking to access two-dimensional (2D) information. This is particularly evident when using 2D tactile readers, devices designed to provide 2D information using static tactile representations with audio explanations. Traditional pinpointing methods, such as sighted assistance and trial-and-error, are limited and inefficient, while alternative pinpointing user interfaces (UI) are still emerging and need advancement. To address these limitations, we develop three distinct navigation UIs using a user-centred design approach: Sonar (proximity-radar sonification), Voice (direct clock-system speech instructions), and Sonoice, a new method that combines elements of both. The navigation UIs were incorporated into the Tactonom Reader device to conduct a trial study with ten BVI participants. Our UIs exhibited superior performance and higher user satisfaction than the conventional trial-and-error approach, showcasing scalability to varied assistive technology and their effectiveness regardless of graphic complexity. The innovative Sonoice approach achieved the highest efficiency in pinpointing elements, but user satisfaction was highest with the Sonar approach. Surprisingly, participant preferences varied and did not always align with their most effective strategy, underscoring the importance of accommodating individual user preferences and contextual factors when choosing between the three UIs. While more extensive training may reveal further differences between these UIs, our results emphasise the significance of offering diverse options to meet user needs. Altogether, the results provide valuable insights for improving the functionality of 2D tactile readers, thereby contributing to the future development of accessible technology.

## 1 Introduction

Two-dimensional (2D) and graphical data are an integral part of our daily lives, starting from our early education years, where we explore educational graphics, to complex visualisations like neural network architectures. However, individuals with blindness or visual impairment (BVI) face significant challenges in accessing and comprehending this visual information. While current assistive technology offers solutions for accessing simple text-based content through screen reader software and single-line braille readers, the accessibility of graphical information remains limited. Graphical elements such as images, graphs, tables, flow charts, formulas, web pages, and floor plans pose significant barriers for individuals with BVI. While tactile printed graphics combined with audio descriptions have been employed, they fall short when presented with complex graphical data that involves numerous elements or dynamic real-time interactions. Addressing these limitations is crucial to fostering equal access and promoting inclusiveness for individuals with BVI in our increasingly visual society.

Emerging technologies have made significant strides in addressing the challenge of providing access to 2D information for individuals with visual impairments (BVI). Tactile graphic readers, coupled with 2D pin-matrix displays, have emerged as promising solutions. Tactile graphic readers integrate tactile information through swell and braille paper with audio feedback, allowing for a dynamic representation of information. In contrast, 2D pin-matrix braille readers combine audio feedback with a grid of refreshable tactile pins distributed over a two-dimensional surface. These technologies have garnered considerable attention, particularly in developing mechanisms for raising and lowering tactile pins (1, 2). However, despite these advancements, there are still numerous obstacles and user interface challenges to overcome, such as the Midas touch effect, information overload, and audio-tactile synthesis representation (3–7). These challenges highlight the ongoing need for dedicated research and development in audio-tactile user interfaces, aiming to enhance the accessibility of 2D information for individuals with BVI.

The pursuit of optimal user interfaces for tactile graphics readers is confronted with a range of intricate and intriguing challenges. Such a significant challenge is assisting individuals with BVI in pinpointing elements on 2D tactile surfaces. This task is of great importance as it allows users to find the starting position of a graphic or engage in focused exploration by locating specific elements or areas within the graphic. However, the task becomes notably demanding when employing audio-tactile user interfaces with large surface sizes. The broader range of possible fingertip positions on these expansive surfaces makes it more challenging for users to pinpoint desired positions and elements precisely. Traditionally, users have relied on the assistance of sighted individuals who guide their fingertips to the desired positions on the tactile surface. However, this approach diminishes the independence of using the technology autonomously. Without sighted assistance, users often resort to the trial-and-error method, consisting of exploring each element individually through tactile textures and audio descriptions. While this strategy fosters free exploration and user autonomy, it becomes difficult to apply in scenarios involving complex graphics with a large number of elements. In such cases, locating a specific element or detail within the information cluster requires significant time and effort, ceasing efficient information retrieval.

Extensive research has delved into diverse methods aiding individuals with BVI in pinpointing elements on tactile surfaces. Beyond the trial-and-error approach, these encompass sonification, speech, and haptic feedback. We conducted an in-depth literature review to understand how individuals with BVI have employed these methods and to explore their main advantages and applications. Since tactile graphic readers are still emerging with limited contributions, our analysis extends to encompass all technologies that deliver graphical information to people with BVI.

### 1.1 Trial and error

Arguably, the trial-and-error strategy is the most common method for people with BVI to locate elements on tactile surfaces. Users explore graphic elements individually until they find the desired element, building a mental representation of the content. On touch screens, users explore elements through speech descriptions (8, 9) or vibration feedback (10). In 2D tactile readers and 3D models, users explore through audio descriptions and braille labels (11–21). This approach supports free exploration but lacks guidance for locating all elements. People with BVI have highlighted the need for an assistive interface to pinpoint elements on tactile surfaces (9, 20–24).

### 1.2 Sonification based

A sonification-based user interface uses sound processing, including tone frequency and gain changes, to guide users to a specific location on a 2D plan. Inspired by the typical car parking aid, one strategy is to use one fixed background sound and increase its frequency as the user gets closer to the target (25). This technology is familiar to users with BVI since it is used in other aid technologies (26, 27) including pinpointing a target rotation direction (28, 29), aiming a camera to the correct angle (30) or for learning line shapes (31). Some strategies use different sounds to map the X and Y axis positions (23). Similar to the car parking aid, the closer the user gets to the correct X or Y position of the target, the higher the frequency of the sound mapped to that axis. While this strategy has the potential to give more details about the target location, it requires that users move their hands in a straight line through the axis, which is a difficult task for people with BVI (22). It is also a common sonification-based strategy in assistive technology for the BVI to create a background that delineates the exact x and y position of the user (32). This aid does not directly guide the user to one element but helps contextualise the user’s current position. Another strategy is to associate a sound with each element on a graphic. The audio is played when the user approaches one of the elements. This approach can also be used with 3D spatial audio, substantially increasing the perception of closer elements (33). Nevertheless, for graphics with many details, the user would be overloaded with multiple sounds from several elements, rendering this approach unreliable for complex graphics.

### 1.3 Speech based

Speech-based strategies use speech instructions such as the cardinal directions or the clock system to guide the user’s hand to a specific position on the tactile surface. Cardinal directions speech strategy uses (top, bottom, left, and right) instructions to guide people with BVI to a specific position on large 2D surfaces. This strategy has been used in touch screens and tactile graphic readers (22, 34), but it is also common in other technological contexts (24, 30, 35, 36). More refined approaches extend beyond directional cues, incorporating proximity feedback through volume adjustment (22) or subtle modifications to speech instructions, such as using “go a little left” instead of “go left” when the user is close in proximity (21). The clock direction system is an alternative to the cardinal system (3 *o’clock*, 6 *o’clock*, 9 *o’clock and* 12 *o’clock*). Some interfaces extend beyond the typical 4-directions to utilise a 12-direction system, providing superior precision in directional guidance, as successfully implemented in technologies such as BlindSquare (37). In (38), the authors concluded that BVI people prefer the clock system to voice instructions when locating elements in indoor floor plans. For others, it is a matter of personalising (39, 40). Some users prefer a clock system, while others prefer voice instructions of the cardinal directions, as some prefer faster and others prefer regular text-to-speech audio speeds (27). Nevertheless, clock system interfaces are common in assistive technology for BVI users (41). Previous research has shown that voice guidance helps BVI people pinpoint and target elements effectively. However, users revealed dissatisfaction with the repetitive and potentially irritating nature of using voice-based feedback (22, 24, 35, 38, 39).

### 1.4 Haptic based

Beyond audio-based strategies, some have used haptic feedback to assist people with BVI in pinpointing elements in tactile surfaces, including extra markers and cutouts, additional wearable tools, and representation changes on the tactile surface. Additional hardware like 3D-printed textural overlays provides quick access but requires replacement if elements move (42). Dynamic magnetic markers offer guidance but lack precision (43). The HyperBraille project’s pin-blinking UI highlights elements but needs a high refresh rate not supported by most 2D pin-matrix readers (44–47). These methods facilitate pinpointing but do not wholly guide the user’s fingertip to the target position. Hand-wearable interfaces offer haptic feedback but negatively affect haptic sensitivity and restrict tactile contextualisation (48–53). Movable guide sliders like the Graille 2D braille display offer precise positioning but limit tactile interaction due to single-finger use (54). 2D refreshable pin-matrix displays, such as those from the HyperBraille project, provide zooming-panning operations that facilitate the location of elements but do not fully guide the user’s finger to the target element (55, 56). Overall, using additional hardware and wearable interfaces to assist people with BVI in pinpointing elements on tactile graphics is not a scalable strategy, working exclusively on the devices that implement each additional hardware. For this reason, we did not develop a solution of this kind in this paper since we were looking for a solution that could be extendable to a more extensive set of technologies (tactile graphic readers, touchscreens, and 2D pin-matrix displays).

Despite the significant number of approaches developed thus far, a standardised solution for pinpointing elements in 2D tactile graphic readers has yet to be established. A sound-based approach seems to be the best option for effective and scalable use in this family of assistive technology. Past research (22) has revealed that a Voice-based is more efficient and effective in assisting users in pinpointing elements in tactile graphics readers. Still, sonification solutions have been considered beneficial in other applications for BVI (29, 57–60). Moreover, combining the advantages of the two approaches is possible, potentially leading to performance benefits. By addressing these issues, the current study aims to contribute to the ongoing discussion on which sound-based approach is the most efficient for pinpointing elements of 2D data.

In this investigation, we address the limitation of element pinpointing within tactile graphics and further investigate potential solutions through a user-centred design approach, closely collaborating with BVI employees from Inventivio GmbH. This collaboration effort led to the development of three unique navigation user interfaces, with two adopting state-of-the-art approaches (Sonar and Voice) and introducing an entirely novel approach (Sonoice). Sonar UI is based on proximity-radar sonification navigation, the Voice UI utilises direct speech instructions with clock-system commands, and the Sonoice UI combines sonification with voice feedback. These UIs were carefully designed to improve the accuracy and efficiency of pinpointing elements, specifically tailored to meet the needs of individuals with BVI. The design choices were based on the widespread adoption of sonification and speech-based UIs in assistive technology, facilitating enhanced access to tactile graphics as supported by relevant studies (9, 22, 34, 50, 61, 62).

Building upon this foundation, our study conducted a comprehensive comparison of the new Sonoice UI with two other previously established audio-based UIs (Sonar and Voice) and the trial-and-error strategy, serving as the baseline benchmark. The Sonoice UI strategy could have been expected to be the most efficient and satisfying method overall as it aims to combine the advantages of the Voice and Sonar UIs. Although the primary objective is the performance of the Sonoice UI, we keep the analysis open and unbiased, i.e., perform a general comparison of all strategies. Thus, we investigate whether these UIs could surpass the trial-and-error approach in effectively guiding users to their desired location. By pursuing this line of inquiry, we aimed to gain invaluable insights into the impact of all user interface strategies, whether they would be more effective in guiding the user to the target location, and recognise the potential complexities that could arise from integrating multiple signals.

## 2 Materials and methods

### 2.1 Participants

The study involved ten participants, four females and six males, who were visually impaired or blind. Participants were recruited from Osnabrück city and its surrounding metropolitan region in north-western Germany. The recruitment process involved close collaboration with the local Lower Saxony blind association BVN, which included distributing accessible documents and featuring an audio segment about our study in their newsletter. Interested individuals who responded to the segment via email were then sent additional information and subsequently participated in the study. Only those who reported a medical diagnosis of visual impairment or blindness were included in the study, as we did not measure visual acuity directly. The University of Osnabrück ethics committee approved the study protocol before recruitment, and informed consent was obtained from all participants after they were briefed about the study’s nature.

While the number of participants does not yet allow for a rigorous statistical analysis of visual impairment subgroups, we have categorised and recorded the results at this level to enable future meta-analyses incorporating data from diverse studies. Based on self-reports, two participants were grouped as congenitally blind (CB), five as late blind (LB), and three as visually impaired (VI) (see Table 1).

**Table 1:** Demographic Data of Participants (P1-P10).

Users	Age range	Gender	VI type	VA level	Experience with 2D UIs
P1	65+	Male	VI: visually impaired	<6/60	No
P2	65+	Male	LB: late blind	<3/60	Navigation aids, braille display
P3	45–64	Female	LB: late blind	<3/60	Navigation aids
P4	45–64	Male	LB: late blind	<3/60	Navigation aids, PC interfaces
P5	65+	Female	LB: late blind	<3/60	No
P6	45–64	Male	VI: visually impaired	<6/60	No
P7	18–45	Female	CB: congenitally blind	<3/60	Applications, visual-tactile aids
P8	18–45	Male	CB: congenitally blind	<3/60	Various navigation UIs
P9	65+	Female	LB: late blind	<3/60	No
P10	45–64	Male	VI: visually impaired	<6/60	Navigation and accessibility UIs

Visual acuity (VA) levels defined by the WHO (63).

Exclusion criteria involved age (under 18), current or past substance abuse, and medical abnormalities that could interfere with the aim of the study, such as those impacting cognitive functions, the sense of touch, hearing or communication disorders, or the motor system. The inclusion criteria for the study involved participants with either an English or German language background. Study materials were provided in both languages as accessible documents or audio recordings. Additionally, none of the participants had hearing or communication disorders.

Due to their “low representation in the general population and mobility difficulties” (64), recruiting participants with BVI for user studies can be a challenging task (33, 58, 65–68). As a result, the number of BVI participants in this study was relatively small. However, involving users and conducting multiple usability tests to follow a user-centred design methodology is crucial. While the small sample size is a limitation, it marks a positive step forward, paving the way for more extensive studies in the future.

### 2.2 Materials

**Figure 1:**
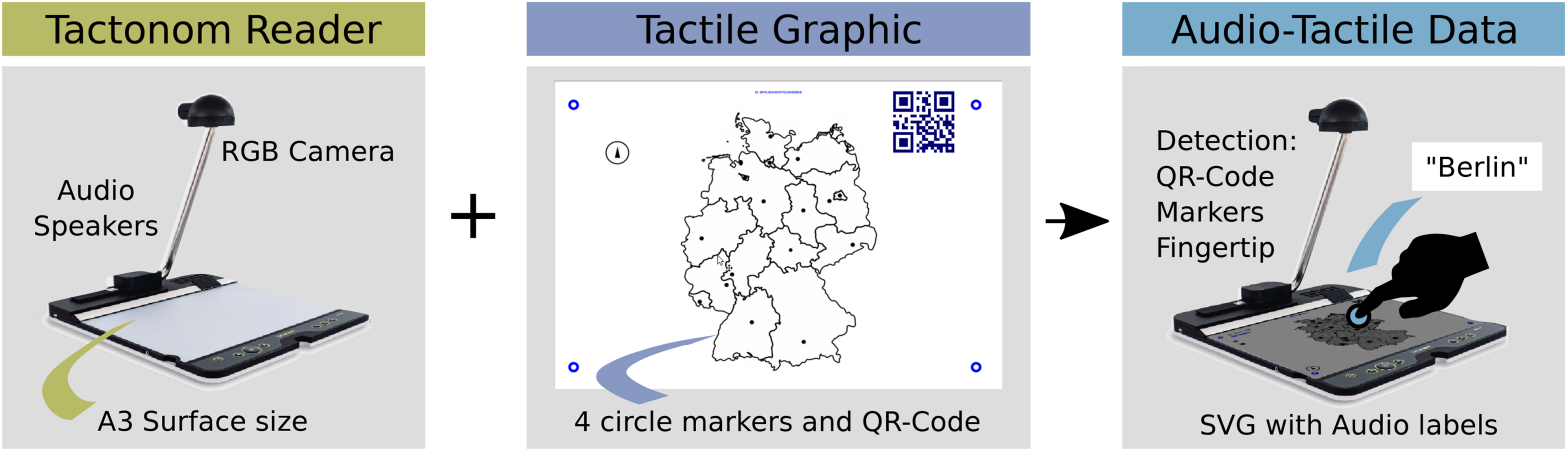
The Tactonom Reader 2.5.0v workflow.

The developed pinpoint strategies were tested and implemented on the Tactonom Reader (Inventivio GmbH) (69), a 5.3 kg tactile graphic reader with a 29 cm by 43 cm magnetic metallic surface (Figure 1). This device integrates tactile graphics (swell or braille paper) with audio explanations, using an RGB camera to detect a QR code that links to an SVG file containing shape elements (<*line*>, <*rect*>, <*circle*>, and <*path*>) and corresponding audio labels. Four corner markers map the SVG elements to the tactile paper on the metallic surface. Fingertip detection via the RGB camera allows users to access audio information by pinpointing graphic elements. Additional details on the Tactonom Reader are in (22, 69). This study used version 2.5.0, released in March 2023.

**Figure 2:**
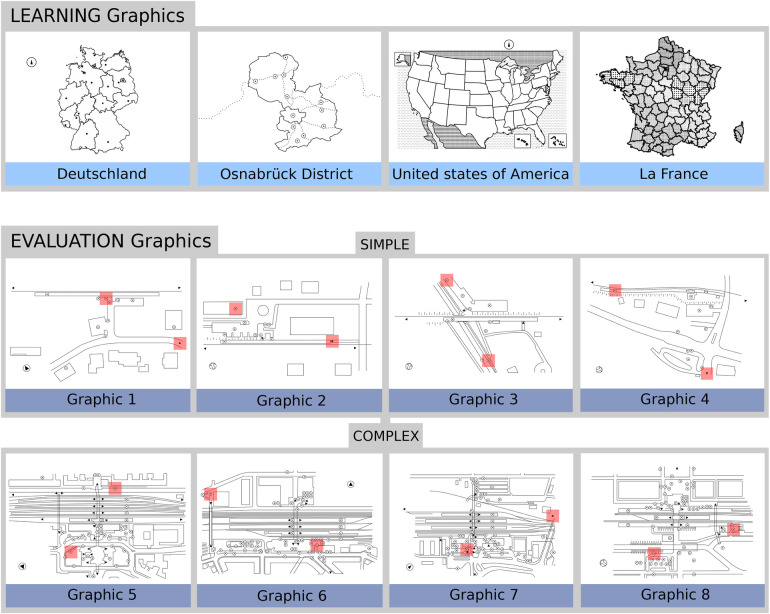
Graphics used to assist participant learning (top) and evaluate the pinpoint navigation strategies (bottom) in this study. The red squares demarcate the target elements participants were required to locate on the evaluation graphics during the testing session. These squares were enlarged to three times their original size to facilitate ease of viewing. For clarity, the blue targets and QR code from the Problind database layout have been intentionally omitted from this figure.

We implemented the pinpoint strategies on the Tactonom Reader using graphics from the open-source Problind database (70), which contains over 3,000 compatible SVG graphics across various contexts, including education, geology, biology, chemistry, mathematics, music, entertainment, and floor plans. For this study, we used four graphics from the Problind database for context exploration and designed eight new SVG graphics for the testing session, all following the Problind layout (Figure 2).

To assist users in understanding and learning the pinpoint navigation strategies, we used four original graphics from the Problind database: *Deutschland*, *Osnabrück District*, *La France*, and *United States of America*, each in their distinct language (German, French, and English). *Deutschland* and *Osnabrück District* were included to offer users familiarity with their regional context. *La France* and *United States of America* were chosen for their popularity and to provide diverse, engaging perspectives while showcasing the customisation and scalability of the Problind database (70). As users with BVI have shown interest in map representations in past studies (22, 71, 72), these graphics were selected to make the interaction and user-interface learning more engaging for participants.

To evaluate the pinpoint navigation strategies, we designed eight graphics representing train station floor plans across Germany, graphics 1 to 8. These are split into two categories: simple train stations and complex train stations. This complexity is expressed by the total number of spot elements, which are small circles and triangles SVG shapes with a square annotation area of 10 mm by 10 mm. The annotation demarcates the region where the fingertip must be positioned to access the additional information. Graphic 1 to 4 are simple train stations with an average number of 14 spot elements per graphic. Graphic 5 to 8 are complex train stations with an average number of 79 spot elements per graphic. Within the spot elements of each graphic, two elements were assigned as the target elements that the user will have to pinpoint in the evaluation session. Beyond the spot elements, the train stations include audio labels on the platforms, train tracks, streets, and outside buildings. The spot elements annotations themselves demarcate points of interest in the train station, including entrances, elevators, bus stops, cafes, information points, bicycle parks, and others. We used train station representations since train stations are among the most visited places by people with BVI (72, 73), and mobility and orientation applications are not as developed as other fields in this emerging technology (7). We designed these graphics and added audio labels using the open-source software *Inkscape* on an SVG blank page with the Problind layout. All SVG elements were rendered in black with a stroke width of 0.5 mm. The completed SVG graphics were uploaded to the database, printed on swell paper, and processed through the PIAF (*Tactile Image Maker*) heating chamber (74). All learning and evaluation graphics used in this study are shown in Figure 2.

### 2.3 Pinpoint navigation strategies

**Figure 3:**
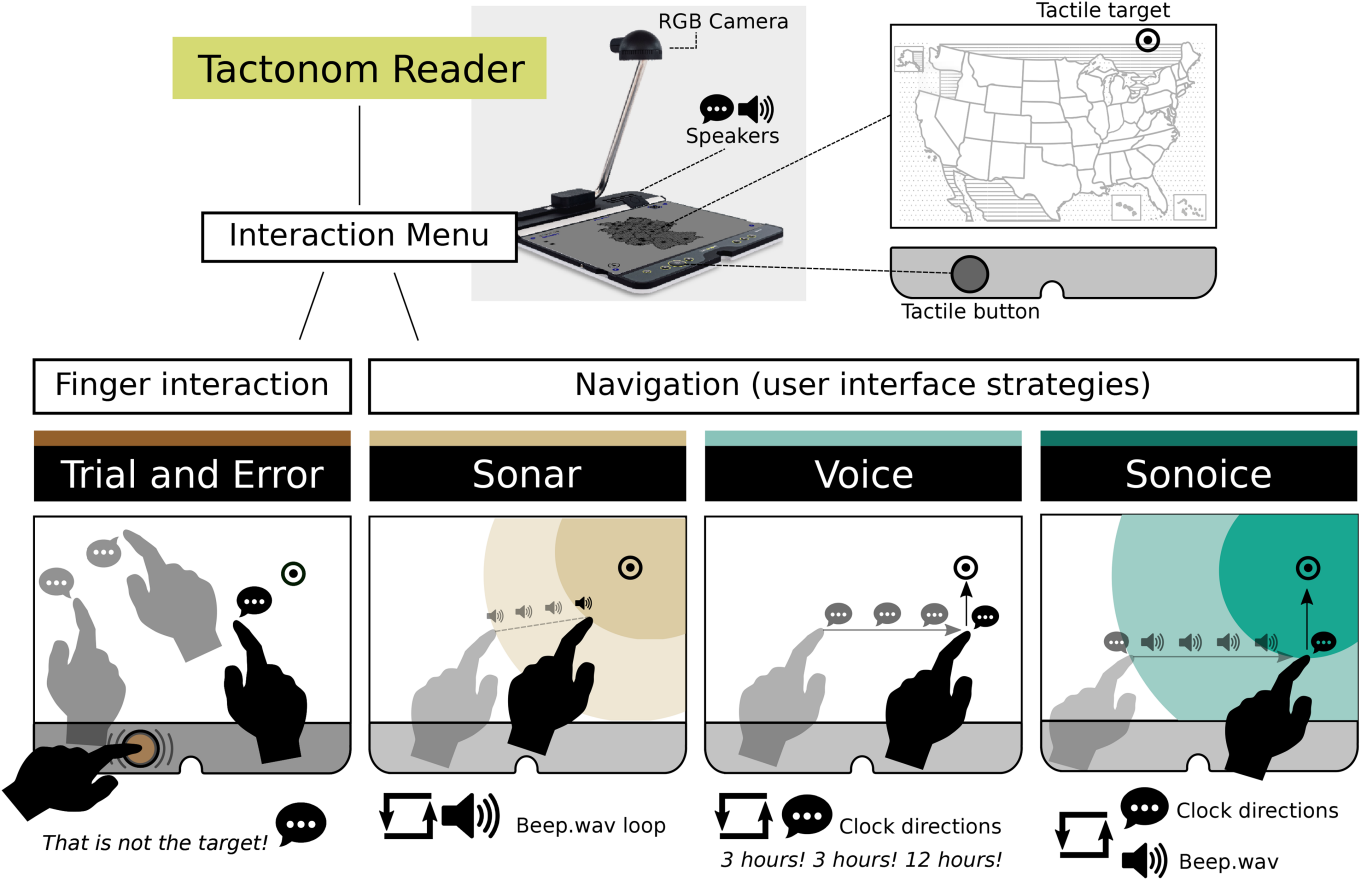
Pinpoint Navigation Strategies for Tactile Graphics (trial-and-error, Sonar, Voice, and Sonoice). The figure presents the workflow and key components of the four navigation strategies used in our study for element pinpointing in tactile graphics. Each strategy is illustrated with a diagram depicting the user’s interaction and associated audio cues.

We explored four pinpoint navigation strategies: Trial-and-Error, Sonar, Voice, and Sonoice (Figure 3). These were implemented on the Tactonom Reader using the MINIM audio processing library version 2.2.2, which handles real-time adjustments in volume, pitch, and panning (75). Our experiments utilised a 100 by 100 digital space to map the user’s fingertip and target location, ensuring consistent audio behaviour across different surface sizes. This digital space allows our audio strategies to be applied to various devices by converting any two-dimensional space accordingly.

Individuals with visual impairments often rely on **trial-and-error** to locate elements on tactile surfaces. This involves exploring each interactive element on a surface one by one until the target element is found. In the context of the Tactonom Reader device, the user can access the information using a combination of hand gestures. Specifically, one hand presses a button while the other serves as a cursor indicator on a 2D tactile graphic. Every time a user queries an element, the device provides audio feedback to indicate the information associated with the element. This information is presented in an audio format, such as text-to-speech or sound. Exploring tactile graphics with a simple button press interface and audio feedback helps to minimise cognitive load and maximise accessibility for individuals with BVI.

Sonification, specifically the **Sonar pinpoint navigation**, is an alternative to the trial-and-error approach for locating elements on tactile surfaces. This strategy draws inspiration from submarine sound navigation and leverages audio feedback to guide users in locating target elements. A background beep sound with a frequency of 412.150 Hz is used to provide auditory feedback, with the frequency and volume of the sound increasing as the user’s fingertip gets closer to the target element. While Sonar navigation had previously been implemented in the Tactonom Reader and introduced in prior research (22), user-centred design has led to significant new improvements to meet user’s needs. We use a linear regression function y=mx+b, where m=−0.0217 and b=2.89 to quantify the magnitude of frequency variation in the beep sound. In this equation, x represents the distance between the user’s fingertip position and the target element in the 100×100 digital space, while y represents the frequency increase of the beep sound relative to its baseline frequency of 412.150 Hz. As the user approaches the target element, the frequency of the beep sound increases. For a distance of 0 in the digital space, indicating that the user’s fingertip has precisely reached the target element, the frequency increase reaches its maximum value of 2.89, corresponding to a frequency of 1,191 Hz (2.89 × 412.150 Hz). The background beep sound has a duration of 0.22 s and is played in a loop while the Sonar navigation is active. All the duration and frequency value adjustments were fine-tuned during user-centred design testing with BVI individuals at Inventivio GmbH.

While sonification is a popular technique for tactile navigation, **Voice pinpoint navigation** offers an alternative method for guiding users towards their target element. This technique involves delivering verbal instructions to the user, indicating the direction of the target element in relation to their fingertip position. Our past research and user-centred design have already looked at voice navigation, where we concluded that direction voices such as “top” and “bottom” caused ambiguity and confusion regarding whether to interpret these cues in a 2D or 3D context (22). Consequently, we implemented a novel variation of Voice navigation, incorporating the clock system, which specifies directions as “3 o’clock,” “6 o’clock,” “9 o’clock,” and “12 o’clock”. Although some successful navigation technologies use additional clock directions like “2 o’clock” or “5 o’clock” (37), we deliberately excluded these from our voice UI, and chose to prioritise simplicity and familiarity, aligning it more closely with the majority of the clock-speech guidance systems used in our context (38–41). While additional directions offer increased precision, they come with the drawback of added processing time and still require micro-adjustments. Past research has concluded that BVI individuals have difficulties pinpointing elements in a straight line along vertical and horizontal directions (22), making diagonal movement potentially more confusing and less efficient for them. The Minim audio library is utilised to adjust the volume of the voice instructions and pan the sound in stereo as the user approaches the target element, providing additional auditory feedback. The specific voice command played is determined based on the biggest distance between the user’s fingertip and the target element. This ensures the voice feedback is consistent and reliable, regardless of the user’s specific starting position on the tactile surface. We used German clock system voices to meet the needs of German-speaking participants in this study.

Following a user-centred design approach to enhance pinpoint navigation speed and user satisfaction, we have developed a novel strategy called **Sonoice** (sonar + voice) that combines sonification and voice pinpoint navigation. Sonoice begins with a single voice direction instruction using the clock system, followed by a continuous loop of a beep sound. The voice direction is determined by the largest distance to the target element, be it vertically (12 or 6 o’clock) or horizontally (3 or 9 o’clock) oriented. As the user approaches the target, the volume and frequency of the beep sound dynamically adjust following the same linear regression function employed in the Sonar navigation strategy. This continues until the user reaches the target element’s x or y threshold based on the voice instruction. For the direction voices “3 o’clock” and “9 o’clock”, this threshold is the x position, while for the voices “6 o’clock” and “12 o’clock”, it is the y position. When the user reaches the threshold, a trigger sound plays and a new voice instruction is given to guide the user towards the target element. Once again, a background beep sound starts playing in a loop until the user reaches the target’s x or y threshold. To enhance user guidance, the Sonoice strategy incorporates a wrong-direction feedback mechanism. If the user moves in the opposite direction of the previous voice instruction, the system replays the last instruction to provide corrective feedback. In addition to addressing the issue of moving the fingertip in a straight direction, which was present in the previous study (22), the Sonoice method offers further usefulness. By continuously giving new voice instructions at each x or y threshold of the target element, the method ensures that the user is always directed towards the target. Additionally, if the user stays still for over 3 s, a new voice instruction is triggered based on the larger distance to the target element. Overall, the Sonoice method attempts to integrate the benefits of both sonar and voice pinpointing strategies, offering a comprehensive and novel approach to tactile surface navigation.

When users lift their hand off the tactile surface of the Tactonom Reader, causing it to go out of view of the camera, the audio feedback is immediately silenced, regardless of the current pinpoint navigation strategy. Upon reaching the target, the system plays a sound to indicate success, “*success.wav*”, and all navigation sounds are stopped and turned off. The stereo sound distribution is enabled for all pinpoint strategies, but due to the Tactonom Reader’s speaker placement, the panning effect may not be noticeable. The Tactonom Reader does not play any other embedded digital audio information during navigation. All methods operate at a 10 FPS rate, corresponding to the RGB camera’s fingertip detection speed, enabling real-time interaction.

### 2.4 Procedure and design

**Figure 4:**
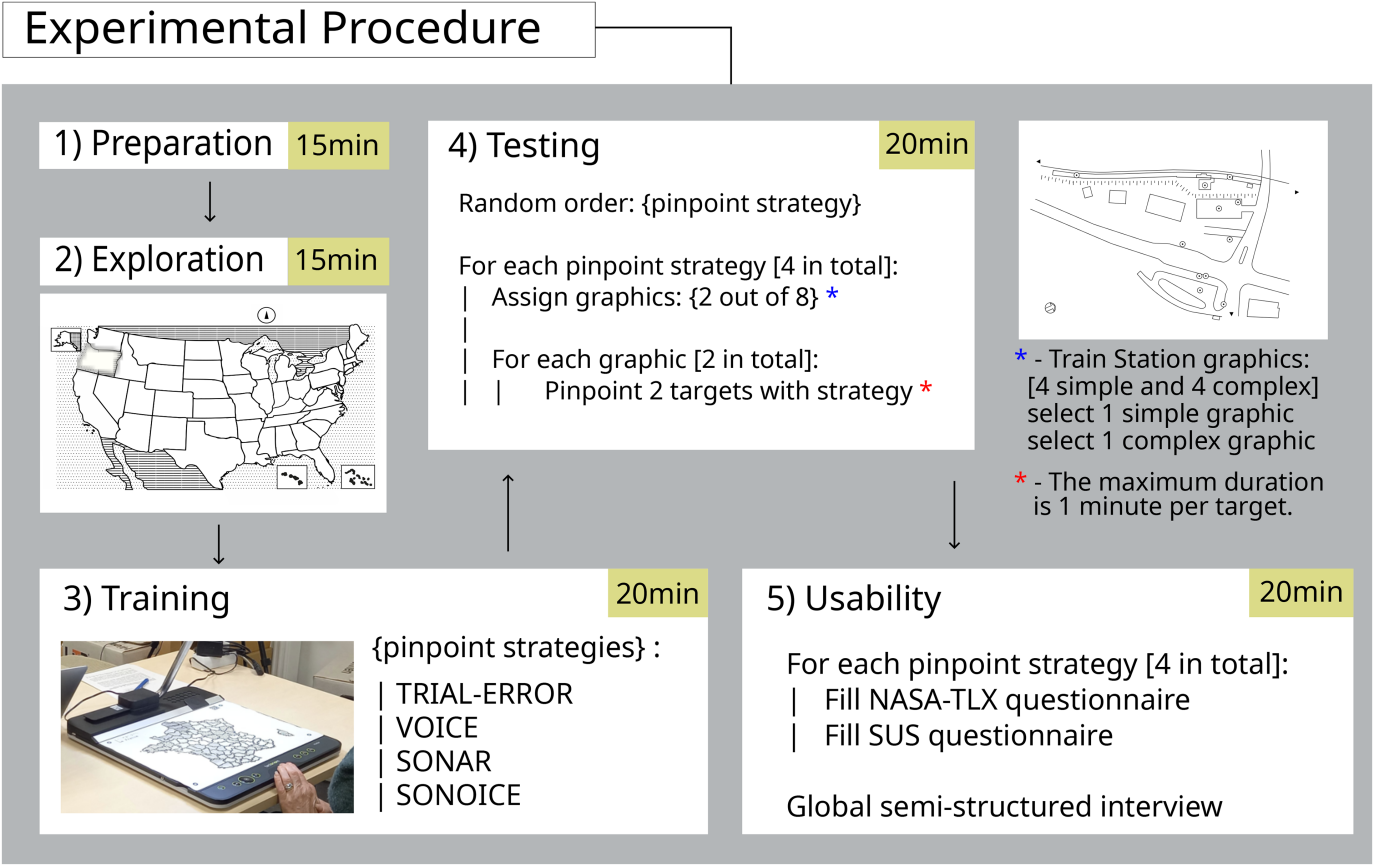
Overview of the experimental procedure phases. After the preparation phase, participants could explore the Tactonom Reader functionalities. All four pinpoint strategies, trial-and-error, Sonar, Voice, and Sonoice, were learned in the training graphic and used to pinpoint targets in 8 train station graphics. The experiment ends with a usability phase, including NASA-TLX and SUS questionnaires and a semi-structured interview.

We employed a within-subjects design for the study, where each participant was randomly assigned to test all four pinpoint strategies. The tests were conducted individually in a single 90-min session for each participant. Figure 4 illustrates the step-by-step progression of the experimental procedure, ensuring clarity and enhancing comprehension of the distinct phases involved.

#### 2.4.1 Preparation

At the beginning of the study, the participants were given a detailed explanation of the study’s purpose and procedures. They were then asked to provide their consent either by signing a consent form or providing a verbal agreement, which was audio recorded. Participants were informed that they could stop the experiment at any time without giving any reason. This phase lasted for 15 min.

#### 2.4.2 Exploration

Subsequently, the exploration phase started, and participants received fifteen minutes to explore the Tactonom Reader with their hands. Beyond getting used to the device dimensions and creating a mental image, participants were allowed to interact with the learning graphics (Figure 2) to understand the Tactonom Reader workflow.

#### 2.4.3 Training

Following the exploration phase, participants underwent a training phase where they learned the four pinpoint strategies implemented on the Tactonom Reader. The training phase began with placing one of the learning graphics on the device. During this phase, participants were instructed to learn all four pinpoint strategies: trial-and-error, Sonar-based, Voice-based, and Sonoice. After selecting an element target from the learning graphic, participants pressed the “enter” button, and a “beep” sound marked the start of the trial. Participants then used the selected strategy to pinpoint the target. They were allowed to repeat the training trials several times for a maximum duration of five minutes per pinpoint strategy.

#### 2.4.4 Testing

After receiving instructions and confirming their understanding of the experimental procedure, participants entered the main experiment phase, the testing phase. Randomly selected by a computer script, one of the four pinpoint strategies and two train station floor plans (one simple and one complex) were presented to the participants. The chosen pinpoint strategy was introduced and displayed on the Tactonom Reader, and the first graphic was placed on the device. Once participants were ready, they initiated the first trial by selecting the navigation mode through the main menu. A target element was randomly assigned from two options, and its name was announced. After a “beep” sound, participants navigated to the correct target location using acoustic feedback from a navigation UI or trial-and-error strategy. After successfully pinpointing the target element, a “beep” sound marked the end of the trial. Each trial had a duration limit of 60 s. If the participant did not successfully pinpoint the target within the allocated time, a “timeout” sound would mark the end of the trial. The participant was instructed to repeat the same procedure for the remaining target element of the current graphic. Once all target elements in the current graphic were located, the second train station floor plan was presented on the Tactonom Reader. The participant then finds two targets on the second graphic using the same strategy. This procedure was then repeated for the other three pinpoint strategies, with their order randomised to eliminate any potential bias. To provide participants with flexibility in using their preferred strategies, they were instructed to place their index finger anywhere on the surface of the Tactonom Reader. The initial position was intentionally not fixed to allow participants the freedom to navigate as they preferred.

#### 2.4.5 Usability

The final part of the experiment involved a NASA-TLX (76) and SUS (77) questionnaire for each pinpoint strategy and an interview that aimed to assess the participants’ user experience. More specifically, it aimed to assess the usability of the Tactonom reader and evaluate how practical the different pinpoint strategies were in guiding a blind or visually impaired user to a particular element in tactile graphics. As we additionally tried to answer the question of what other aspects of the Tactonom Reader and the implemented strategies could be improved, the interview was conducted as a semi-structured interview. This allowed the experimenter to ask additional questions in case the participant reported intriguing observations next to the general questions that were the same for all participants (available in the Supplementary Material).

### 2.5 Data analysis methods

We employed a mixed-methods approach, integrating both quantitative and qualitative data, including interviews. The analyses of the behavioural data, including UI performance and the impact of graphical complexity, use total trial times as the dependent variable (to assess efficiency), employing parametric statistics (ANOVA) for statistical testing. Questionnaires (NASA-TLX and SUS) are evaluated using standard normalised scores (to assess user satisfaction), while subjective data from interviews and open-ended questions are documented with descriptive statistics and illustrated using original user quotes.

## 3 Results

Our investigation aimed to assess the efficiency and user satisfaction of four distinct navigation strategies (Figure 3) employed for pinpointing elements in 2D tactile graphics. To accomplish this, we conducted a comprehensive analysis of quantitative and qualitative data obtained during the testing and usability phases, thereby providing a thorough validation of the diverse pinpoint strategies. Although we did not perform further statistical analysis on these subgroups, the results include data categorised into the three types of visual impairment: CB (congenitally blind), LB (late blind), and VI (visually impaired). Henceforth, participants’ comments will be accompanied by their identifier, type of visual impairment, and favourite navigation strategy (e.g., P7, CB, Sonar).

The results of our study are presented across four key sections. Section 3.1 compares the efficiency of the four navigation strategies (trial-and-error, Sonar, Voice, and Sonoice). Section 3.2 analyses user satisfaction while using these strategies, and Section 3.3 examines the differences in performance when interacting with simple or complex tactile graphics. Additionally, Section 3.4 investigates the distinct fields and contexts to which these UIs can be applied.

### 3.1 Efficiency analysis of pinpoint navigation strategies

**Figure 5:**
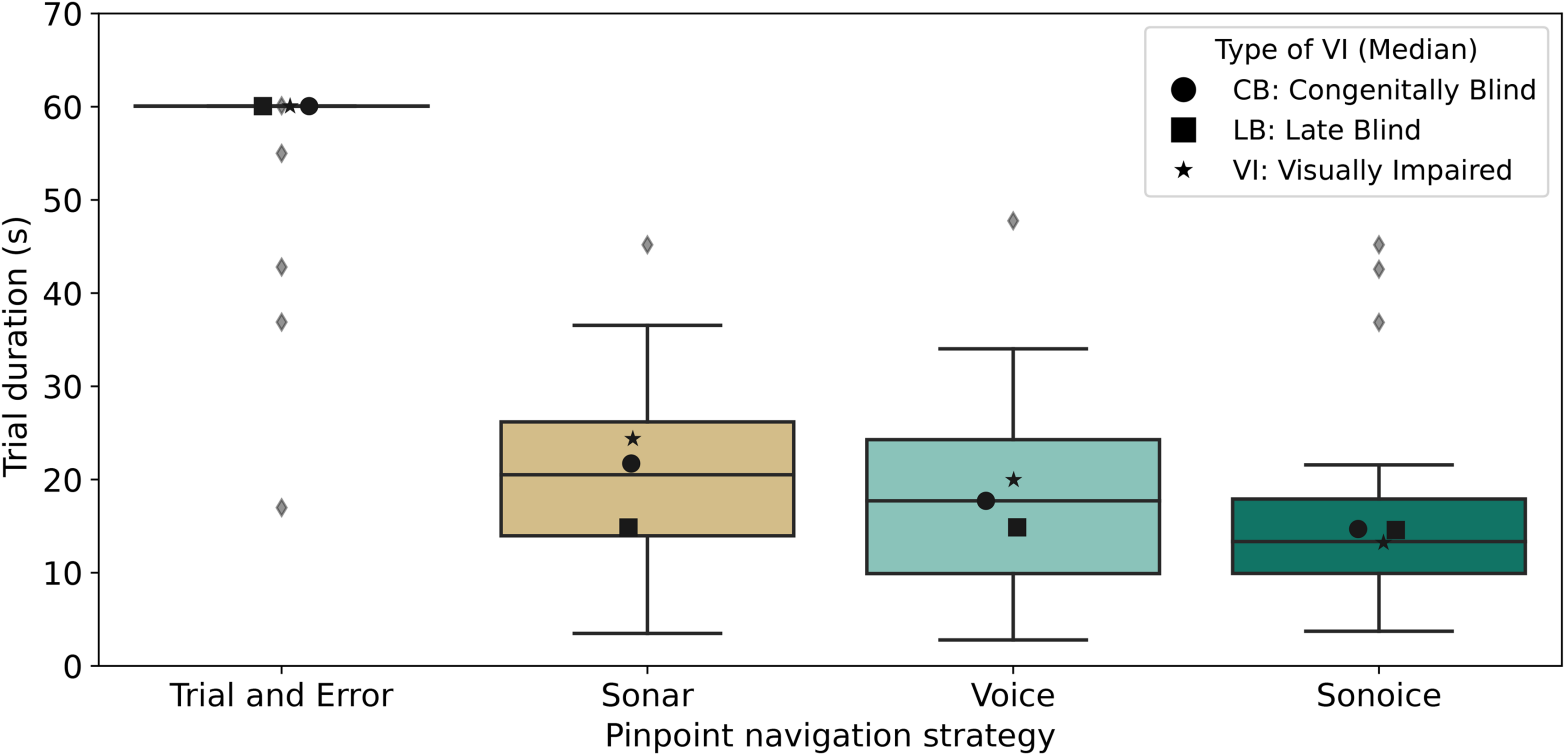
Comparison of Pinpoint Navigation Strategies based on trial duration. Boxplots show the distribution of trial durations for each strategy (in seconds), with medians represented as solid lines. Outliers are depicted as grey diamonds. The black markers denote the medians of each subgroup of visual impairment type for each boxplot distribution: circles for *CB*, squares for *LB*, and stars for *VI*.

To conduct a comparative analysis of the four pinpoint navigation strategies (trial-and-error, Sonar, Voice, and Sonoice), we began by examining the distribution of trial duration in seconds for each strategy. The mean elapsed time required by participants to locate the target element was 57.85±8.04 s for trial-and-error, 20.68±8.99 s for Sonar, 17.58±9.50 s for Voice, and 15.48±8.91 s for Sonoice (Figure 5). Notably, among the 40 trials conducted using the trial-and-error approach, only four trials (10%) were successfully completed within the designated time limit of 60 s, while the remaining trials reached the maximum duration allowed (Figure 5).

Employing a repeated measures ANOVA statistical test with α=0.05, we aimed to assess whether there were any significant variations in mean trial durations across different strategies. Our results showed a statistically significant difference between the mean trial durations of the four strategies (F(3,27)=139.5827,p<0.001). The calculated F-value (139.5827) exceeded the critical F-value (2.9604) for the test, leading us to reject the null hypothesis. These findings indicate a significant difference in the mean trial durations among the four pinpoint navigation strategies.

To determine the specific nature of the differences between the navigation strategies, pairwise t-tests were performed on the average trial duration for each strategy. The results revealed significant differences between several pairs of strategies. The trial strategy exhibited substantial differences compared to the Sonar (t=−12.83,p<0.001), Voice (t=−18.00,p<0.001), and Sonoice (t=−22.78,p<0.001) strategies, indicating that the trial strategy was significantly less efficient than the other three. However, we found no significant differences between the Sonar and Voice methods (t=−1.12,p=0.291) or between the Voice and Sonoice methods (t=1.22,p=0.255), or the Sonar and Sonoice methods (t=−1.95,p=0.083).

Interestingly, the Sonoice method exhibited consistently lower mean trial durations than the other strategies, although statistical tests did not yield significant differences. While these findings suggest the potential efficiency of Sonoice in pinpointing elements in tactile graphics, further data would be necessary to determine whether this effect reaches statistical significance.

### 3.2 User-satisfaction analysis

To assess user satisfaction with the various pinpoint navigation strategies, we employed subjective measures, including NASA-TLX and SUS questionnaires, along with semi-structured interviews.

**Figure 6:**
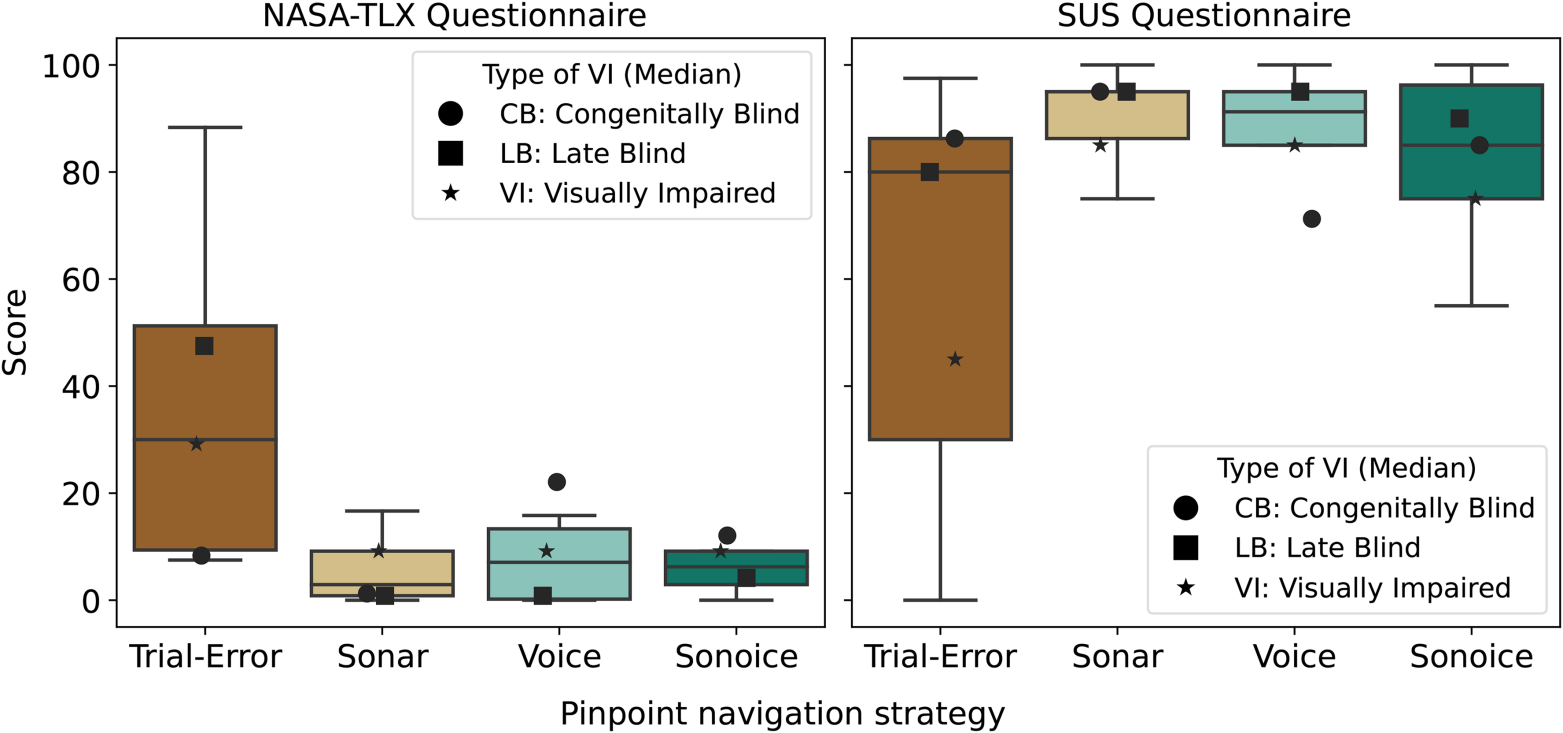
Comparison of subjective workload and satisfaction ratings across pinpoint navigation strategies. The left plot shows the NASA-TLX scores, while the right plot shows the SUS scores for the trial, Sonar, Voice, and Sonoice strategies. The black markers represent the medians of each subgroup of visual impairment type for each boxplot distribution: circles for *CB*, squares for *LB*, and stars for *VI*.

The NASA-TLX and SUS questionnaires were administered to each participant after they completed the navigation tasks with each strategy. The NASA-TLX questionnaire, measured on a scale of 0 to 100, assesses subjective workload, with lower scores indicating reduced cognitive load. Similarly, the SUS questionnaire, measured on a scale of 0 to 100, evaluates overall satisfaction, with higher scores representing greater user satisfaction. Results from the NASA-TLX questionnaire showed that the mean scores (± standard deviation) for the Trial-Error, Sonar, Voice, and Sonoice strategies were 33.67±26.90, 5.50±5.95, 10.00±13.45, and 8.75±9.21, respectively (Figure 6). These results suggest that the trial strategy may have imposed a higher workload on the participants since its average score is at least three times bigger than any other navigation strategy. To understand if there was any significant difference between the user-interface strategies for pinpoint elements (Sonar, Voice, and Sonoice), we performed a repeated measures ANOVA statistical test with α=0.05. Results indicated no substantial disparity in the mean NASA-TLX score across the navigation strategies (F(2,18)=0.394,p=0.983), suggesting that these are equally effective regarding overall user workload.

Regarding the SUS questionnaire, results showed that the mean scores (± standard deviation) for the Trial-Error, Sonar, Voice, and Sonoice strategies were 59.75±36.39, 88.50±13.95, 84.00±17.96, and 83.25±14.67, respectively (Figure 6). The trial strategy had the lowest mean SUS score, indicating it was the least satisfactory method overall. The other three strategies all received an average score not only above the average (68) but above 80, which is considered a high score by past research (78, 79). These results suggest that participants rated the Sonar strategy as the most satisfactory, followed by the Voice and Sonoice strategies. To determine if there were any significant differences between the user-interface strategies for pinpoint elements (Sonar, Voice, and Sonoice), we performed a repeated measures ANOVA statistical test with α=0.05 on the SUS scores. The results showed no significant difference between the strategies (F(2,18)=0.780, p-value = 0.473).

**Figure 7:**
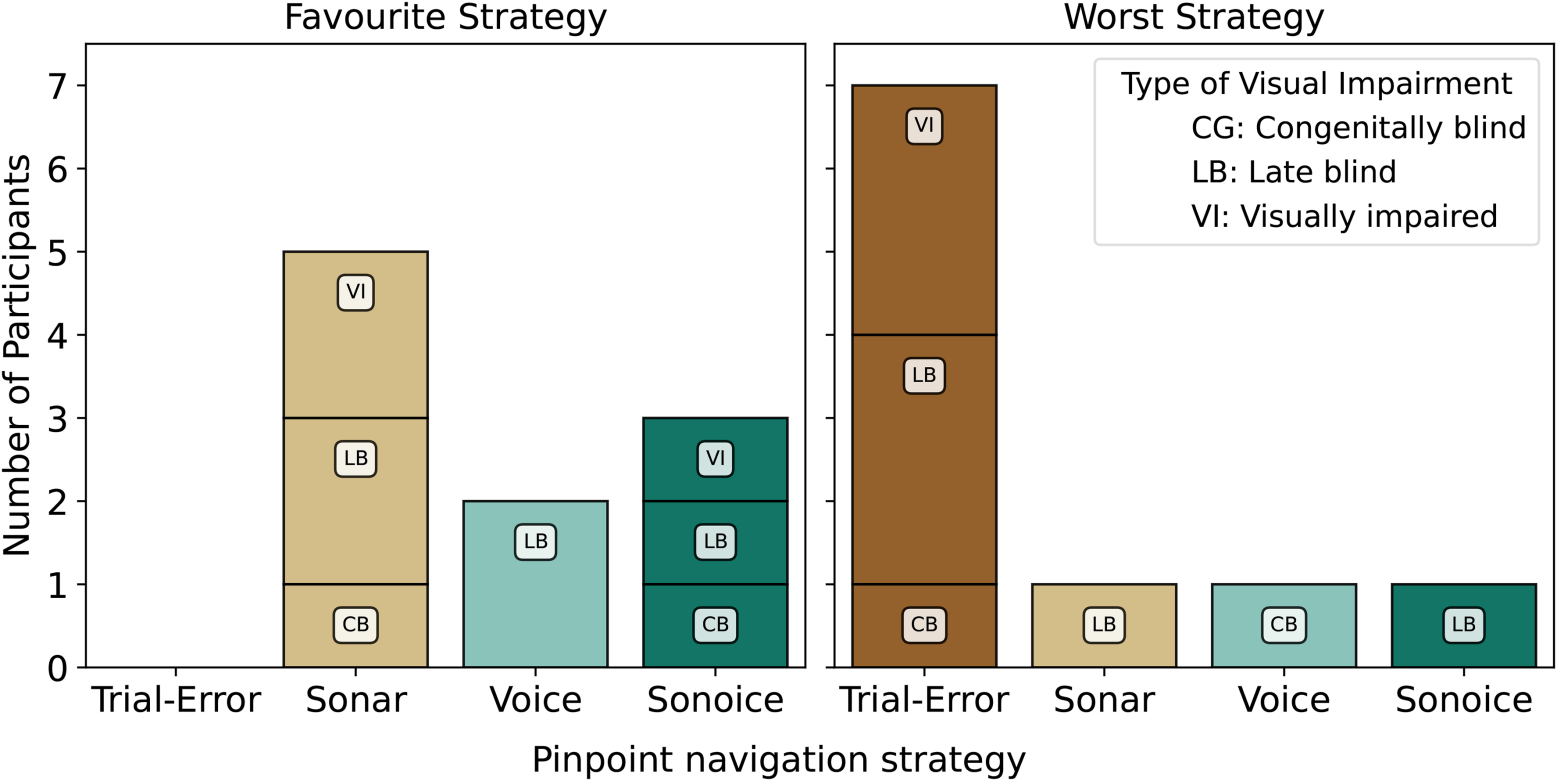
Distribution of favourite and worst pinpoint strategies reported by participants (10 in total) during semi-structured interviews. Each bar chart is segmented by the type of visual impairment, denoted by CB, LB, and VI votes.

Although neither NASA-TLX nor SUS questionnaire results showed significant differences between the Sonar, Voice, and Sonoice strategies, it is important to note that these are subjective measures and may not capture all aspects of user satisfaction. Therefore, it is still important to consider the valuable qualitative feedback obtained from the semi-structured interviews. In the interviews, participants were specifically asked about their most and least favourite strategies for pinpointing elements in tactile graphics (Figure 7). This additional insight allows us to gain a deeper understanding of participants’ preferences and experiences with the different pinpoint strategies.

We proceeded to analyze the specific remarks provided during the usability session to acquire further insights not only into the overall subjective evaluation but also to elucidate the underlying rationale behind this decision.

Among the participants, the Sonar interface emerged as the most highly rated strategy, receiving a total of 5 out of 10 votes as the favourite choice (Figure 7). Participants provided positive feedback regarding the Sonar strategy, highlighting its familiarity, responsiveness, and intuitive nature: “*The Sonar because it uses a principle that I am familiar with and it feels more responsive and more intuitive*” (P7, CB, Sonar), “*Sonar (voices are difficult to hear when there are other people around). It is well distinguishable from natural sounds*”(P10, VI, Sonar), “*Sonar because it is super quick and intuitive!*” (P4, LB, Sonar), “*My favourite was Sonar, but Sonoice is still a great option although it uses a lot of information which can confuse you!*” (P1, VI, Sonar).

The Sonoice user interface was the second most highly rated strategy, receiving 3 out of 10 votes as the favourite choice (Figure 7). Participants recognised the benefits of utilising a combination of sound and voice to obtain more detailed information and accurately pinpoint the target position: “*Sonoice is direct guidance combined with fast guidance. With more information, you get there faster! It depends a bit on how well you’re able to multitask, but it has high potential!*” (P6, VI, Sonoice), “*Most of all, Sonoice because it first provides the general direction and then more fine-tuned details!*” (P8, CB, Sonoice), “*Sonoice because you get a much better overview of the environment in general and the spatial relationships.*” (P2, LB, Sonoice).

Additionally, a subset of participants (2 out of 10) preferred the Voice user interface (Figure 7). These participants found the Voice method to be straightforward to use and responsive: “*The Voice method is very specific and straightforward!*” (P3, LB, Voice), “*The Voice since it is directly interpretable and can change quickly.*” (P9, LB, Voice).

Contrarily, the trial-and-error strategy consistently received the least favourite rating, with 7 out of 10 participants expressing dissatisfaction (Figure 7). Feedback regarding the trial strategy highlighted limitations, such as uncertainty, feeling helpless, and tediousness. Participants shared comments like “*Just with trial and error, you are limited! I feel helpless and don’t know what to do! It is uncomfortable and feels more like a TOY than a tool.*” (P3, LB, Voice), “*It is tedious to press the button constantly in the trial and error approach*” (P1, VI, Sonar), and “*The trial and error strategy is difficult to apply in the context of finding an element! Requires a lot of time and pressing!*” (P5, LB, Sonar). Despite its drawbacks for pinpointing elements, participants recognised the trial-and-error strategy’s usefulness for obtaining an overview of the graphic content, as expressed in statements like “*The worst was trial-and-error to localise but to explore it’s amazing! It should be the first step to explore with this mode to get an overview*” (P8, CB, Sonoice) and “*The trial-and-error strategy would be ideal for exploring as part of mobility training!*” (P2, LB, Sonoice).

Each of the remaining three navigation strategies received one vote as the least favourite, with participants pointing out their specific drawbacks (Figure 7). Some participants expressed challenges with the Sonar strategy, mentioning the difficulty in realising they were moving in the wrong direction, “*Sonar was the worst! It took me super long to change directions and to realise when I was going in the wrong direction. I could not react quickly enough to avoid going in the wrong direction.*” (P9, LB, Voice). The Voice strategy was criticised for requiring excessive mental effort in interpreting the clock system, “*Voice is the worst because I needed to think too much about the clock and where the 3 h is located!*” (P7, CB, Sonar). Participants also found the Sonoice strategy overwhelming, as it demanded sustained concentration, “*Sonoice is too much, and concentration is hard to keep!*” (P4, LB, Sonar).

Based on the results of the NASA-TLX, SUS, and semi-structured interviews, the three user interface strategies for pinpointing elements in tactile graphics have demonstrated their usefulness, exhibiting statistically higher satisfaction levels compared to the standard trial-and-error approach. All ten users unanimously agreed that they found at least one of the three navigation pinpoint user interfaces more useful than the trial-and-error method for locating elements in tactile graphics. Furthermore, all participants highly recommended the navigation user interfaces to other individuals with BVI, “*I absolutely prefer the navigation modes, and I think the Tactonom with these would be a great addition to my current devices!*” (P10, VI, Sonar), “*I would use them. I would retrieve much more information from the graphics with the navigation strategies!*” (P8, CB, Sonoice).

Our analysis revealed that while the Sonoice UI received positive feedback from participants, we did not gather sufficient evidence to conclude that it consistently outperformed the other strategies regarding user satisfaction. It is worth noting that participants’ preferences and experiences varied across the different navigation strategies, and no significant differences were found in overall user satisfaction between the Sonar, Voice, and Sonoice strategies according to the collected data.

In summary, the findings indicate that the implemented user interfaces significantly improve user satisfaction compared to the traditional trial-and-error approach. Based on these results, we conjecture that these navigation strategies hold a large potential to enhance the accessibility and usability of tactile graphics for individuals with BVI. Further research and larger sample sizes may be necessary to explore potential differences in satisfaction among the various pinpoint navigation strategies in more detail.

### 3.3 Unveiling the influence of graphic complexity

**Figure 8:**
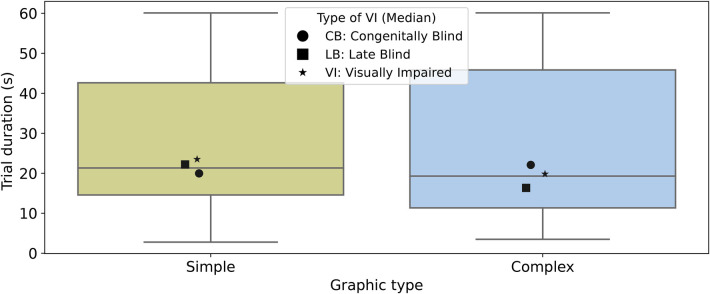
Distribution of trial durations (s) per graphic type (Simplex and Complex). The boxplot displays the medians as solid lines. Black markers represent the medians of each subgroup of visual impairment type for each boxplot distribution: circles for *CB*, squares for *LB*, and stars for *VI*.

To fully understand the efficiency of different navigation strategies in tactile graphics, it is important to explore the impact of graphic types on the performance of these navigation strategies. To address this, our comprehensive analysis covered both simple and complex graphics. The analysis aimed to assess the potential disparities in element pinpointing performance between the two graphic types. Surprisingly, the results revealed no significant difference in the mean trial duration between complex graphics (27.10±20.28 s) and simple graphics (28.69±18.86 s) (Figure 8). These findings challenge our initial assumptions and suggest that graphic complexity does not significantly impact the time required for pinpointing elements. Importantly, this lack of difference holds true across the navigation pinpoint user interfaces (Sonar, Voice, and Sonoice) and the trial-and-error approach.

**Figure 9:**
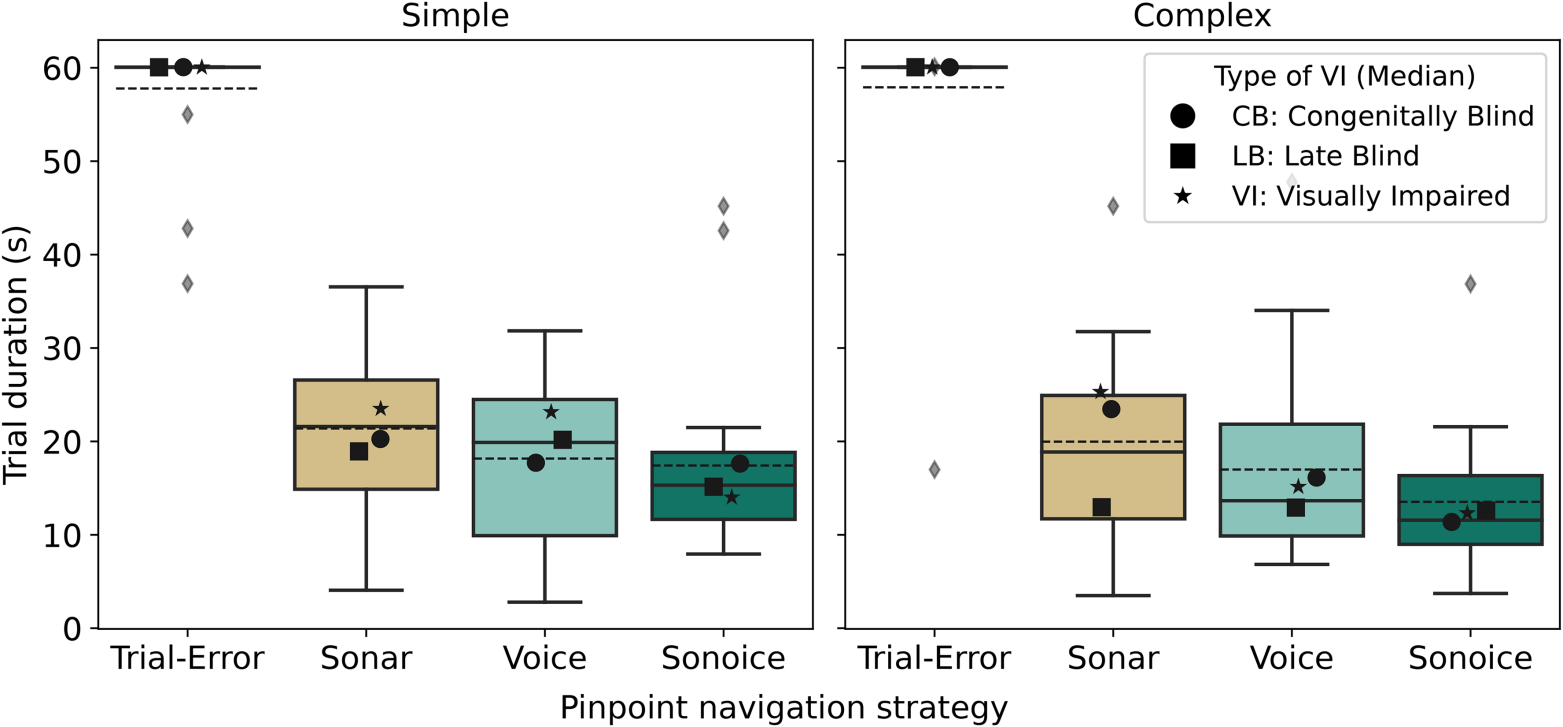
Distribution of trial durations (s) across graphic type (Simple and Complex) and pinpoint navigation strategy. The boxplot displays the medians as solid lines, while the dashed lines represent the means. Outliers are depicted as grey diamonds. Black markers represent the medians of each subgroup of type of Visual Impairment (VI) for each boxplot distribution: circles for *CB*, squares for *LB*, and stars for *VI*.

In addition to evaluating the performance of different navigation strategies on simple and complex graphics, we comprehensively analysed the data using a boxplot to visualise the average trial durations across the graphic type and pinpoint strategy (Figure 9). Subsequently, we aimed to test whether a navigation user interface (UI) allows people with BVI to pinpoint elements in complex graphics more efficiently than the trial-and-error strategy. The results demonstrate the superiority of the Sonar, Voice, and Sonoice navigation strategies over the trial-and-error approach for complex and simple graphics. In complex graphics, the mean trial duration was 19.98±9.85s for Sonar, 16.99±10.39s for Voice, and 13.53±7.60s for Sonoice, while the trial-and-error approach had a significantly higher mean trial duration of 57.91±9.63s. Similarly, in simple graphics, the mean trial duration was 21.39±8.25s for Sonar, 18.16±8.75s for Voice, and 17.42±9.86s for Sonoice, compared to 57.79±6.31s for the trial-and-error approach (Figure 9).

These findings provide strong empirical evidence that a navigation UI enables individuals with BVI to pinpoint elements in both complex and simple graphics more efficiently compared to the trial-and-error strategy. We interpret these results to suggest that the UIs might be successfully applied beyond complex graphics and underscore their potential to improve accessibility and usability across a wide range of tactile graphics with varying complexity.

### 3.4 Expanding applications of pinpoint navigation interfaces

In addition to locating elements in floor plans in tactile graphics, pinpoint navigation strategies offer broader applications. During the semi-structured interviews, participants shared their perspectives on the contextualisation of pinpoint navigation interfaces in various fields of assistive technology. Their comments revealed diverse potential uses, including emergency floor plans, schools, public services, navigation apps like the Seeing AI App, country maps, and even household appliances like washing machines where specific settings can be easily located, “*In floor plans or countries maps. It would be amazing to use it in washing machines and find a certain setting.*” (P5, LB, Sonar). Participants also highlighted the advantage of complementing pinpoint navigation with on-site sensor-based navigation technologies (37, 59, 60, 80) like the FeelSpace naviBelt: “*Use navigation modes for practical preparation and then the FeelSpace belt for mobile applications.*” (P10, VI, Sonar). The idea is to use pinpoint navigation for mobility training, developing mental representations of changing environments, and preparing for visits and trips, followed by on-site navigation aids for real-time assistance.

It was found that 7 out of 10 participants had never interacted with a similar technology beyond the Tactonom Reader device itself, indicating that this technology is still emerging and not readily accessible to users. These subjective evaluations by the users highlight the novel and evolving nature of pinpoint navigation interfaces, underscoring their potential for future applications in various domains and assistive technology.

## 4 Discussion

Our investigation into the efficiency and user satisfaction of various navigation strategies in tactile graphics has yielded significant insights, contributing to improving tactile information access. Notably, all tested pinpoint user interface strategies outperformed the trial-and-error approach, demonstrating their superiority in facilitating efficient pinpointing of tactile elements. Among these strategies, the Sonoice UI, which combines auditory and voice cues, emerged as the most efficient. However, satisfaction ratings were surprisingly deviant from performing ratings. Participant’s feedback shed light on this phenomenon, stating, “*My favourite was Sonar, but Sonoice is still a great option although it uses a lot of information which can confuse you!*” (P1, VI, Sonar), “*Sonoice is too much, and concentration is hard to keep!*” (P4, LB, Sonar). This contrasting perspective adds complexity to the relationship between performance and user satisfaction, emphasising the need for a comprehensive understanding of user preferences and subjective experiences when pinpointing elements in tactile graphics.

### 4.1 Balancing performance and user satisfaction

Exploring both performance and user satisfaction across all navigation strategies uncovered intriguing insights, defying the conventional notion that the most efficient method would necessarily be the most favoured. Interestingly, except for the trial-and-error approach, which yielded anticipated results, the remaining strategies yielded unexpected outcomes.

#### 4.1.1 High performance

This unexpected divergence was particularly evident in the performance of the Sonoice method. Despite not receiving user satisfaction ratings as high as the Sonar method, the Sonoice method exhibited the lowest mean trial duration during the experiments. This raises the question: how could Sonoice achieve higher efficiency despite slightly lower satisfaction ratings? The answer may reside in the combination of advantages of the Voice and Sonar approaches. The Voice method provides directional guidance but lacks information on the distance to the target and can be confused with natural sounds “*voices are difficult to hear when there are other people around*” (P10, VI, Sonar) (57, 62). On the other hand, the Sonar strategy offers proximity feedback but requires users to interpret pitch sound differences to ensure they are moving in the right direction. With the Sonoice method, we aimed to combine the advantages of both the Sonar and Voice strategies, leveraging their strengths to create a more effective approach. By incorporating directional guidance from the Voice method and proximity feedback from the Sonar method, we sought to provide users with a comprehensive and efficient navigation experience, “*Sonoice is direct guidance combined with fast guidance. With more information, you get there faster!*” (P6, VI, Sonoice). Notably, recent studies have shown that assistive interfaces incorporating both sonification and voice feedback jointly have yielded promising results (28, 40, 81–83), suggesting that combining sonar with voice can possibly enhance the effectiveness of tactile graphics exploration.

#### 4.1.2 High user satisfaction

Although it emerged as the most efficient method, the Sonoice method was not the most preferred strategy during the task. An explanation for this is that Sonoice uses more information than the other two methods, which some users saw as overwhelming, “*My favourite was Sonar, but Sonoice is still a great option although it uses a lot of information which can confuse you!*” (P1, VI, Sonar). Another factor that could have contributed to this may stem from the fact that assistive technology typically relies on either voice or sonification approaches (9, 34, 50, 61, 84), making a combination of these two methods less common and potentially leading to unfamiliarity or hesitation among users.

The theme of familiarity and user preference is further underscored in the case of the Sonar approach. Despite not being the fastest approach, Sonar obtained the highest satisfaction rate, possibly influenced by participants’ experiences with assistive technology. Participant comments substantiate this connection, as seen in statements like “*The Sonar because it uses a principle that I am familiar with*” (P7, CB, Sonar) and “*Sonar because it is super quick and intuitive!*” (P4, LB, Sonar). These findings underscore the influence of participants’ prior experiences and contextual factors in shaping their preference for a particular navigation UI, aligning with similar observations in related research (29, 58).

Regardless, it’s worth noting that participants received only 5 min of training for each strategy. With extended training, users would potentially become more familiarised and less overwhelmed with the Sonoice approach, changing the results of this investigation. Moreover, these potential changes are also subject to individual differences and the type of visual impairment each participant has.

### 4.2 The value of the trial and error approach in tactile graphics exploration

While being the least favoured approach and the least efficient in pinpointing elements in tactile graphics, the trial-and-error method still holds value for users. Despite not being ideal for precise element identification, this approach proves to be beneficial for initial exploration and gaining a contextual understanding of the graphic. It allows users with visual impairments to familiarise themselves with the layout and content of the graphic, providing a starting point for further interaction and interpretation. In fact, this method is implemented in other 2D tactile graphic readers (11–18, 20, 23), highlighting its significance in facilitating exploration and providing an overview. As one participant remarked, “*The worst was trial-and-error to localise, but to explore it’s amazing! It should be the first step to explore with this mode to get an overview*” (P8, CB, Sonoice) While the trial-and-error method may not provide direct and precise guidance to pinpoint elements, it can contribute to the overall understanding of the two-dimensional information presented.

Given its value in facilitating initial exploration, the trial-and-error functionality should be included in assistive technology for tactile graphics. By recognising its role and benefits, developers can ensure that users with visual impairments can access a range of strategies that cater to different aspects of their exploration needs, enhancing their overall experience and access to 2D information.

### 4.3 Assessing complexity in train station floor plans

In assessing complexity in train station floor plans, results revealed that the choice of navigation UI strategy (Sonar, Voice, and Sonoice) did not yield significant differences in performance between simple and complex graphics. This indicates that our user interface strategies demonstrated consistent effectiveness regardless of the complexity of the tactile graphic. However, the trial-and-error approach presented a different outcome, as most of the samples reached completion within the given time limit. It is worth considering that if the trial duration had not been restricted to 1 min, we might have observed contrasting results using the trial-and-error method for simple vs. complex graphics. These findings are particularly intriguing, as they shed light on the time-consuming nature of interacting with seemingly “simple” graphics, highlighting the inherent challenge individuals with visual impairments face in accessing and comprehending two-dimensional information (85).

## 5 Conclusion

The rapid advancements in 2D tactile readers and 2D pin-matrix displays hold immense potential for revolutionising information accessibility for individuals with visual impairments. One crucial aspect of their usability lies in developing effective user interfaces that enhance the precise pinpointing and locating of elements on 2D tactile surfaces, empowering users to access graphical information independently. Our study has unequivocally demonstrated the superiority of an audio-based navigation user interface approach over the conventional trial-and-error method, thereby significantly improving the accessibility of graphical information for individuals with visual impairments. Significantly, our findings unveiled that our user interfaces (Sonar, Voice, and Sonoice) exhibited exceptional performance in terms of efficiency and garnered excellent user satisfaction ratings. Remarkably, these outcomes were achieved even though participants received only a brief 5-min training session, and some had no prior experience with 2D tactile readers. These compelling results not only shed light on the capabilities of sonification/speech navigation user interfaces but also emphasise the importance of user-centred design in creating inclusive technology for the visually impaired population.

Based on the results and discussions of our study, the Sonoice navigation user interface has emerged as a notable solution, achieving higher levels of efficiency compared to the sonar and voice methods. Remarkably, users achieved these impressive results with just 5 min of training, and many of them quickly recognised the potential of Sonoice, interpreting it as “SO NICE!”. Interestingly, the most efficient method was not the most favoured one. The simultaneous use of sonification with speech feedback negatively impacted the Sonoice method. A combination of methods that should and partly does outperform the other simpler combinations was not appreciated by all users. Some participants found the Sonoice UI information overwhelming compared to the other navigation user interfaces, which, although slightly slower, still performed greatly. Users are willing to trade off some speed in performance for ease of use and to avoid information overload, interpreting Sonoice more as “SO NOISE!” than “SO NICE!” The choice of using one of three navigation user interfaces is highly influenced by participants’ personal preferences and prior experiences. Therefore, understanding individual preferences and tailoring the user interface accordingly is essential for optimising user satisfaction and effectiveness in tactile graphics exploration. In response to these findings, we have integrated all three audio user interfaces, including Sonar, Voice, and Sonoice, into subsequent software updates of the Tactonom Reader.

Our findings highlight the potential of navigation strategies to enhance the accessibility and usability of tactile graphics for individuals with visual impairments, emphasising the importance of incorporating such user interfaces in future design and development efforts. Moreover, our navigation user interfaces can be extended beyond tactile graphics readers and integrated into various technologies, including tablets and 2D refreshable pin-matrix displays. This broader application of navigation strategies contributes to advancing assistive technology in these emerging devices. As tactile graphics readers and 2D refreshable braille hardware technology continue to grow, it is essential to define optimal user interface standards and expand the capabilities and application domains, further empowering individuals with visual impairments.

## Data Availability

The original contributions presented in the study are included in the article/Supplementary Material, further inquiries can be directed to the corresponding author.
