# Supplementary material for: SONOICE! a Sonar–Voice dynamic user interface for assisting individuals with blindness and visual impairment in pinpointing elements in 2D tactile readers
Source: Front Rehabil Sci. 2024 Aug 23;5:1368983. doi: 10.3389/fresc.2024.1368983 (PMC11377411; doi:10.3389/fresc.2024.1368983)
Supplement: Supplementary file 1 [file Datasheet1.pdf]

Semi-structured Interview:
PINPOINT NAVIGATION INTERFACES USABILITY TEST

This interview contains statements regarding the user experience during the usability study phase 1, which includes the interaction with the trial-error approach and the voice interface in the Tactonom Reader. We will fill in the document based on the user's personal experience. Additional questions and answers will be added to this document report as well. The interview will also be recorded with an audio recorder to document the conversation without disrupting the flow.

The interview will be semi-structured and conducted after the experimental usability test session 1 with the Tactonom Reader device. During this usability test phase, the participants might have a special interaction with or an interesting reaction to the device. This will have an influence on the semi-structured interview; additional questions regarding these interactions will be asked.

1. Navigation user interface usefulness:

a) Was the navigation user interface approach more useful in pinpointing elements in tactile graphics than the trial-and-error strategy?

b) Would you use one of the navigation user interfaces or recommend it to others? *If your answer was no*: Do you think there is a general use/application for these or a comparable user interface that helps localize single elements in complex tactile graphics?

c) In what context would you use the navigation user interface and why?

d) Could it be extended to other assistive technologies you use daily?

e) Have you ever interacted with similar user interfaces in other assistive technologies?

2. Favorite method:

a) What was your preferable element-location method?

b) Why was this element-location method your preferred method?

3. Worst method:

a) What was your least preferred element-location method?

b) Why was this element-location method your least preferred method?
   a. If applicable: Did it not work properly? Was there not enough training? Were the elements selected particularly difficult to localize?

4. Experience:

a) Does your preferred strategy depend on the type of graphic? (i.e., for a floor plan, it might be one, but for a table sheet graphic or a diagram, it might be different)

b) After having experience with these techniques, would you still go with your usual approach, or would you use these new algorithms if they are available?

Additional questions regarding specific interactions or reactions during the usability test phase will be added here based on the observations obtained during the experiment.
